# Metabolic flexibility is impaired in response to acute exercise in the young offspring of mothers with type 2 diabetes

**DOI:** 10.14814/phy2.14189

**Published:** 2019-09-08

**Authors:** Timothy D. Allerton, Brian A. Irving, Guillaume Spielmann, Stefany Primeaux, Dennis Landin, Arnold Nelson, Neil M. Johannsen

**Affiliations:** ^1^ Adipocyte Biology Pennington Biomedical Research Center Baton Rouge Louisiana; ^2^ School of Kinesiology Louisiana State University Baton Rouge Louisiana; ^3^ Human Genomics Pennington Biomedical Research Center Baton Rouge Louisiana; ^4^ Department of Physiology Louisiana State University Health Science Center New Orleans Louisiana; ^5^ Preventative Medicine Pennington Biomedical Research Center Baton Rouge Louisiana

**Keywords:** High‐intensity interval training, lipolysis, metabolic flexibility, oxidation, substrate

## Abstract

We assessed metabolic flexibility (MF) via a mixed meal in a group of young, healthy participants with a positive family history of maternal type 2 diabetes (T2D) (FH+) and those without a family history of T2D (FH−) under three distinct conditions; baseline (BL; no previous exercise), 1‐h post high intensity interval exercise (1H), and 48‐h post exercise recovery. On separate visits, participants completed a single bout of high intensity interval exercise (HIIE) and repeated the MMTT 1‐h (1H) and 48 h (48H) postexercise. FH+ participants were not able to suppress fat oxidation 1‐h post exercise (1H) as effectively as FH− participants were, however, this response was improved when measured at the 48H visit. Insulin AUC was significantly lowered at both 1H and 48H when compared to the BL visit. Serum NEFA AUC was elevated 1‐h post exercise, when compared to BL, but was significantly reduced at the 48H visit. Young, healthy participants with a maternal history of T2D demonstrate impaired MF (related to the inability to suppress fat oxidation) in response to acute HIIE (1H) that was improved 48H. The overall effect of HIIE showed improved insulin AUC and NEFA AUC up to 48H post that did not differ by FH.

## Introduction

Type 2 diabetes (T2D) is associated with impaired metabolic flexibility (MF) and vascular dysfunction (Kelley and Mandarino, 2000). The inability to switch efficiently between fuel sources when transitioning between postabsorptive and postprandial stages (metabolic inflexibility) can be explained, in part by differences in glucose disposal rate (Galgani et al. 2008a) and the capacity to increase nonoxidative glucose disposal (Dube et al. 2014). Additionally, the capacity for insulin to suppress lipolysis, and thereby fat oxidation, in the postprandial period is critical for glucose uptake and MF (Horowitz et al. 1997; Galgani et al. 2008a; Sparks et al. 2009). Substrate transport in the postprandial state is, in large part, mediated by the vascular actions of insulin to increase microvascular blood flow (Baron et al. 2000). Insulin‐mediated vasodilation has been shown to be impaired in the presence of elevated FFA by reducing nitric oxide production (Steinberg et al. 2000), and therefore may provide an additional mechanism for FFA‐mediated metabolic inflexibility.

The risk for developing T2D is significantly increased in individuals with a family history (first degree relative) of T2D (FH+) (Rothman et al. 1995; Valdez 2009). Healthy, normo‐glycemic individuals with a positive family history of T2D have been shown to have early signs of insulin resistance, metabolic inflexibility, impaired endothelial‐mediated vasodilation, and mitochondrial dysfunction (Petersen et al. 2004; Goldfine et al. 2006; Hojbjerre et al. 2010; Russell et al. 2013). While chronic exercise training and weight loss have been shown to improve metabolic flexibility to glucose, the response to a mixed‐meal is less clear (Meex et al. 2010; Huffman et al. 2012). Those with a family history of T2D have been shown to be particularly sensitive to deleterious effects of sedentary behavior and bed‐rest, however, little is known about the effects of acute exercise in this at‐risk population (Alibegovic et al. 2009; Hojbjerre et al. 2010).

The purpose of this study was to assess in a group of young healthy participants with a positive family history of maternal T2D (FH+) and those without a family history of T2D (FH−) under three distinct conditions; (1) baseline (BL; no previous exercise), (2) 1‐h post high intensity interval exercise (1H), and (3) 48‐h post exercise recovery. We hypothesized that FH+ participants would be less metabolically flexibile in response to a mixed meal and that acute exercise would improve the MF response.

## Research Design and Methods

This study was approved by Louisiana State University’s Institutional Review Board and participants were provided written informed consent prior to their participation. Seventeen apparently healthy, active, normal weight (BMI = 18–25 kg/m^2^), young (age 19–25 y) adults (6M/11F) participated in this study. Table 1 provides details of the distribution between FH− and FH+ participants. All participants reported being moderately physically active (3 days week of 30 min of moderate intensity physical activity) and none reported engaging in regular high intensity interval training. Individuals with known cardiovascular, T2D, and/or metabolic disorder were excluded from the study. FH+ was confirmed if biological mother of participant was positive for T2D.

**Table 1:** Participant Characteristics.

Status	*n*	Gender	Age (y)	BMI	VO_2peak_ (mL/kg/min)
FH−	9	5M/4F	21.2 + 1.5	23.2 + 2.3	32.2 + 4.7
FH+	8	2M/6F	21.0 + 0.9	26.6 + 2.9	32.3 + 4.4

FH−, no family history of T2D; FH+, mother with T2D.

mean ± SD.

### Study design

After an initial screening, eligible participants completed a graded exercise test on a cycle ergometer (Velotron, Racermate, Inc., Seattle, WA) with indirect calorimetry (TrueOne, ParvoMedics) to determine peak oxygen consumption (VO_2 Peak_). At least 72 h after their VO_2Peak_ test, the participants completed their baseline, resting, mixed‐meal with indirect calorimetry as described below. At least 7 days later, the participants returned for two additional a study visits. The first visit included a single bout of HIIE, as described below, followed by a liquid mixed‐meal tolerance test (MMTT) with indirect calorimetry that was initiated 1‐h post HIIE (1H). The second visit included a follow‐up MMTT with indirect calorimetry that was initiated 48 h post HIIE (48H).

### Mixed meal tolerance test and indirect calorimetry

Prior to the baseline MMTT visit, participants were asked to refrain from exercise, alcohol, and caffeine for 48 h and to provide a record of food intake the day preceding the test. The morning of the test, participants arrived fasted and provided a 24‐h food record. Upon arrival, participants started a 30‐min rest period.

After the 30‐min rest period, the 0‐min resting energy expenditure (REE) measurement was made using a ventilated hood system to determine the volumes expired oxygen (O_2_) and carbon dioxide (VCO_2_) (ParvoMedics Inc., Sandy, UT). Exhaled air was continuously measured for 30 min before the mixed meal (420 kcal, 58 g CHO, 14 g PRO, 14 g Fat) (−30 to 0 min) and following the MMTT (30, 60, 90, and 120 min post‐MMTT) assessments while the participants remained still and refrained from sleeping. The last 10 min of each collection period was averaged and used to report REE, RER, and substrate oxidation rates. REE, carbohydrate (CHO) and fat oxidation rates were calculated utilizing the following equations (11) where REE (kcal/day) = ((3.58 × VO_2_) + (1.448 × VCO_2_)−0.002)) × 1440, CHO Oxidation (g/min) = (4.55 × VCO_2_) − (3.21 × VO_2_) and Fat Oxidation (g/min) = (1.67 × VO_2_) − (1.67 × VCO_2_).

After completion of the 0 min REE measurement, the mixed meal drink (Ensure, Abbott laboratories, Lake Forest, Illinois) was consumed over a period of less than 5 min. Blood was drawn through an IV catheter immediately before the ingestion of the mixed meal and 5, 10, 15, 20, 30, 60, 90, 120, 150, 180 min post‐MMTT. All procedures for the MMTT, including the energy content (kcal) of the mixed meal, were replicated for the 1H and 48H post exercise visits.

### High intensity interval exercise and 48H follow‐up

On a separate week from the baseline measurements, participants arrived fasted overnight having replicated the diet 24 h prior to the baseline MMTT. Participants were also asked to refrain from alcohol, caffeine, and vigorous exercise 48 h prior to the visit. Upon arrival participants completed 30 min of rest followed by the baseline REE measurement. Participants then completed a 5‐min warm‐up on the cycle ergometer (Velotron, Racermate, Inc., Seattle, WA) at 50 watts. Immediately after the warm‐up participants then completed alternating intervals of Watts corresponding to 90% of the watts achieved at peak oxygen consumption during the VO_2Peak_ test for 60 sec followed by 60 sec at 30% of the max watts for a total of 20 min (i.e. 10 sets of interval/rest periods).

After completion of the HIIE intervention participants returned to the laboratory to rest for 60 min (1H), after which fasted, postexercise blood samples were drawn, the mixed meal was provided and sampling proceeded as detailed previously. Forty‐eight hours after the HIIE visit (48H) participants returned to the laboratory to complete the final MMTT of the study. Participants were asked to replicate their baseline study diet and refrain from alcohol, caffeine, and additional exercise between the HIIE visit and 48‐h follow‐up.

### Laboratory analysis

Blood samples were collected in sodium fluoride/oxalate and EDTA tubes and immediately placed on ice. An additional set of silicon coated tubes was used for the collection for serum. The tubes were centrifuged at 450 *g* for 10 min thereafter plasma and/or serum were transferred to plastic microtubes and frozen at −80°C. The glucose oxidase technique was used during the MMTT via a bedside glucose measurement system (GL5, Analox Instruments LTD. Lunenburg, MA). The measurement of plasma insulin was completed with the use of an enzyme‐linked immunoabsorbance assay (ELISA) kit (Sigma Aldrich, St. Louis, MO). Serum non‐esterified fatty acids (NEFA) were quantified by an enzymatic colorimetric assay (Wako chemical, Richmond, VA).

### Statistical analysis

All statistics were performed using JMP version 13 statistical software (SAS Institute Inc., Cary, NC). Data analysis generally followed CONSORT recommendations using General Linear Models and repeated measures analysis of variance (RM‐ANOVA), co‐varied as needed depending on normality distributions at baseline. In addition, two‐way RM‐ANOVAs (visit × time point) were used to determine differences in responses in both the primary and secondary outcome variables. Significant effects were further evaluated using Tukey’s post‐hoc analyses when appropriate. Data are reported as mean ± SD unless otherwise noted. Significant differences were declared at *P* < 0.05.

## Results

### Metabolic flexibility

**Figure 1:**
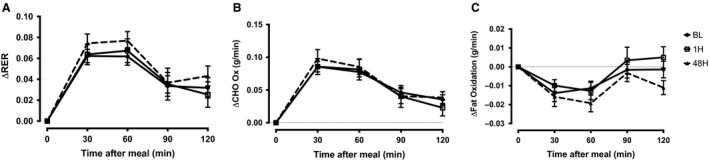
Time course of (A) △RER (RER Postmeal – Premeal RER), (B) △CHO oxidation and (C) △fat oxidation for BL, 1H, and 48H visits. CHO, carbohydrate (g/min), BL, baseline‐ no previous exercise; 1H, 1 h post‐exercise; 48H, 48 h postexercise for all study participants.

**Figure 2:**
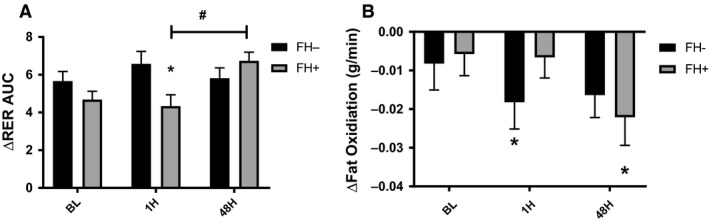
(A) △ RER AUC and (B) △fat oxidation (fat oxidation 60‐min – fasting fat oxidation) during BL, 1H, and 48H comparing FH− to FH+. (*<0.05 different compared to fasting value). FH−, no first or second degree relative with type diabetes; FH+, mother with type 2 diabetes. (*P < 0.05 different between FH− vs. FH+) (^#^ *P* < 0.05).

Figure 1 provides a time course analysis of the measured ΔRER values from premeal to 120‐min postmeal for all visit and participants. There were no statistically significant differences between baseline (5.2 ± 1.3) and 1H (5.3 ± 1.8) or 48H (6.2 ± 1.4) post‐HIIE (all *P* > 0.05). The FH+ group did significantly (*P* = 0.04) improve MF when comparing the 1H versus 48H visit. Since fat oxidation is typically reduced in response to insulin stimulation, we were interested in the degree suppression of fat oxidation. The suppression of fat oxidation at 60 min post meal (Fig. 2) was significantly (*P* = 0.03) attenuated during the 1H visit in the FH− participants (−0.018 ± 0.01 g/min), but not in FH+ participants (0.007 ± 0.01 g/min). During the 48H visit there was a significant improvement in suppression of fat oxidation within the visit (*P* = 0.01), but missed statistical significance (*P* = 0.09) when compared across visits (1H vs. 48H).

### Glucose, insulin, and fatty acids

**Figure 3:**
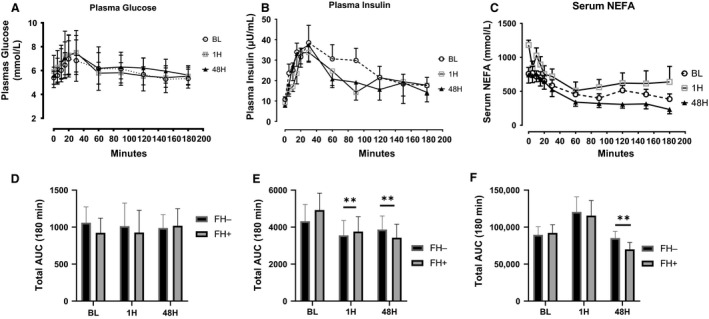
Postprandial plasma glucose (A and D) plasma insulin (B and E) and serum NEFA (C and F) response for BL, 1H and 48H visits in response to a liquid mixed meal. (**P < 0.01 different compared to BL). AUC, area under curve; NEFA, Non‐esterified fatty acids; BL, baseline; 1H 1‐h postexercise; 48 H, 48 h postexercise.

Compared to the baseline visit (5.3 ± 0.5 mmol/L), the plasma glucose measured post‐HIIE premixed meal was increased during the 1H (5.9 ± 1.3 mmol/L, *P* = 0.043) visit when but not the 48H visit (5.5 ± 0.7 mmol/L; *P* = 0.45). Fasting plasma insulin was not different between BL (10.7 ± 4.3 *µ*L/mL) and 1H (9.3 ± 3.3 *µ*L/mL; *P* = 0.68) and the 48H (7.9 ± 3.1 *µ*L/mL; *P* = 0.25) visits or between 1H and the 48H (*P* = 0.73) visits. Fasted serum NEFA levels were significantly greater (*P* = 0.006) at 1H (1.2 ± 0.2) when compared to baseline (0.76 ± 0.2 mmol/L). Fasted NEFA levels returned to near baseline fasted levels at 48H (0.74 ± 0.3). There were no differences in fasting plasma glucose, insulin or NEFA when comparing FH− to FH+. The time course of plasma glucose (Fig. 3A and D), plasma insulin (Fig. 3B and E), and FFA (Fig. 3C and F) demonstrated significant reduction insulin AUC during 1H and 48H visit when compared to BL. Serum NEFA levels were elevated during the 1H visits, but not significantly. During the 48H visit there was a significant reduction in NEFA AUC when compared to BL for all participants. We did not detect any significant difference in glucose, insulin, or NEFA AUC between FH− and FH+ participants.

## Discussion

The primary findings of this study were that healthy, normal BMI, normo‐glycemic adults whose mother had T2D (FH+) had an attenuated MF response to mixed meal after a single bout of high‐intensity interval exercise that was improved 48 h postexercise. The ability to effectively suppress lipolysis and fat oxidation in the postprandial state is considered a critical component of MF and glucose disposal (Singhal et al. 2002; Galgani et al. 2008a; Sparks et al. 2009). Data from healthy volunteers shows that circulating fatty acids are elevated and fat oxidation is increased immediately post exercise, but decreases to sedentary level in response to a mixed meal (Long et al. 2008; Short et al. 2012). The FH− group in our study effectively reduced postprandial fat oxidation 1H post exercise, whereas the FH+ group remained elevated. This suggests that the influx of FFA, a result of intense exercise, attenuated the ability to switch from fat oxidation to carbohydrate oxidation in those FH+ participants.

We did not detect any difference in glucose excursion in response to the mixed meal. The overall carbohydrate content of the meal was 55 g and therefore it is possible that glycemic response is different when higher glucose loads are administered (75 g OGTT). However, others have demonstrated that insulin secretion is synergistically increased with other macronutrients to promote glucose disposal and the use of mixed meal is more physiological that a OGTT (Short et al. 2012). We did measure significant improvements in insulin AUC at both the 1H and 48H visit when compared to BL. Additionally, NEFA levels fell significantly 48H post exercise when compared to BL. These responses were not different between FH− and FH+ participants. The improvement in adipose sensitivity to insulin to reduced lipolysis was paired with a greater ability to increase ΔRER and reduce fat oxidation in the postprandial state. Based on the present data, it would appear that the adipose response to exercise may be mediated by improvements in MF 48H post exercise in FH+ participants.

Previous studies have also demonstrated a reduced response to exercise in those with FH+ status (Irving et al. 2011; Russell et al. 2013). Individuals with FH+ display early signs of metabolic inflexibility (Russell et al. 2013), which likely predisposes them to develop skeletal muscle insulin resistance (Ukropcova et al. 2007). However, Galgani et al. (2008a) demonstrated no impairment in MF in T2D when adjusted for glucose disposal rate. The capacity of insulin to increase vasodilation and postprandial microvascular blood flow accounts for 40–50% of insulin‐stimulated glucose disposal (Bradley et al. 2013; Keske et al. 2016). The role of skeletal muscle microvascular and adipose tissue blood flow could also be a regulator of MF Steinberg et al. (2000), which was not measured in the current study.

The strengths of this study include the use a mixed meal to represent normal physiological feeding to measure MF in two distinct population (FH− vs. FH+). The extended time‐course (48H) postexercise is also a unique strength of this study. A limitation of this study was the inability to assess the level of glycogen depletion during HIIE and to determine if MF was associated with the degree of depletion. The rate of glycogen storage is a primary determinant for MF (Galgani et al. 2008b; Faerch and Vaag 2011) and insulin sensitivity (O'Gorman et al. 2000; Pascoe and Gladden, 1996). The determination of factors involving the time frame of glycogen resynthesis after exercise and in the day after hold considerable potential. The number of calories and carbohydrate in the mixed meal may have been another potential limitation. It is possible that a greater total amount may have produced a response that could be evaluated under the influence of exercise.

Despite our small sample size, the current study provides evidence that young, healthy FH+ participants have an attenuated MF in response to acute high intensity exercise that is restored 48H post exercise. Future studies may be needed to determine the role of microvascular adipose tissue blood flow in the coordination of postprandial inhibition of lipolysis and reduction in fat oxidation.
